# Associations of Nutritional Behavior and Gut Microbiota with the Risk of COVID-19 in Healthy Young Adults in Poland

**DOI:** 10.3390/nu14020350

**Published:** 2022-01-14

**Authors:** Paweł Jagielski, Edyta Łuszczki, Dominika Wnęk, Agnieszka Micek, Izabela Bolesławska, Beata Piórecka, Paweł Kawalec

**Affiliations:** 1 Department of Nutrition and Drug Research, Institute of Public Health, Faculty of Health Sciences, Jagiellonian University Medical College, 31-066 Krakow, Poland; beata.piorecka@uj.edu.pl (B.P.); pawel.kawalec@uj.edu.pl (P.K.); 2 Institute of Health Sciences, Medical College of Rzeszow University, 35-959 Rzeszów, Poland; eluszczki@ur.edu.pl; 3 The Cracow’s Higher School of Health Promotion, 31-158 Krakow, Poland; dsiedlec@cm-uj.krakow.pl; 4 Department of Nursing Management and Epidemiology Nursing, Jagiellonian University Medical College, 31-007 Cracow, Poland; agnieszka.micek@uj.edu.pl; 5 Department of Bromatology, Poznan University of Medical Sciences, 42 Marcelińska Str., 60-354 Poznań, Poland; ibolesla@ump.edu.pl

**Keywords:** COVID-19 pandemic, dietary intake, Dietary Inflammatory Index, gut microbiota, nutrition, physical activity

## Abstract

The numerous consequences of the coronavirus disease 2019 (COVID-19) pandemic in healthy young people and the lack of clarity as to the long-term disease outcomes have spurred the search for risk factors for SARS-CoV-2 infection. We aimed to evaluate the associations of nutritional behaviors, gut microbiota, and physical activity with the risk of COVID-19 in healthy young nonobese people. Data on body composition, anthropometric measurements, physical activity, dietary intake, and gut microbiota were obtained from 95 adults (mean age, 34.66 ± 5.76 years). A balanced diet rich in vegetables and fruit, including nuts, wholegrain cereal products, and legumes, covers the need for vitamins and minerals. Such a diet can be an effective measure to reduce the risk of COVID-19 in nonobese healthy physically active young people with normal immune function. People with balanced diet and an average daily consumption of >500 g of vegetables and fruit and >10 g of nuts had an 86% lower risk of COVID-19 compared with those whose diet was not balanced and who consumed lower amounts of these products. It is well documented that proper nutrition, physical activity, and maintenance of normal weight facilitate good health by ensuring optimal immune function. The beneficial effects of these interventions should be strongly emphasized during the COVID-19 pandemic.

## 1. Introduction

Severe acute respiratory syndrome coronavirus 2 (SARS-CoV-2) and the disease it causes, coronavirus disease 2019 (COVID-19), have changed the world since the beginning of 2020. In March 2020, the US Centers for Disease Control and Prevention presented a report on COVID-19 morbidity, including the age group of 20–44 years [1,2]. At the same time, this age group was reported to account for 20% of hospitalized patients with COVID-19, of whom 12% required admission to the intensive care unit [3]. While young adults typically show a less severe course of the disease compared with older people, they also develop symptoms, especially in the long term. Therefore, there is an increasing concern about COVID-19 symptoms in this age group [4]. In the COVID Symptom Study [5], 13% of patients with COVID-19 (mean age, 43 years) experienced symptoms for more than 28 days. These symptoms may include headache, loss of taste or smell, fatigue, cough, muscle pain, cognitive disturbances, and low-grade fever [4,5,6]. Apart from the health sequelae of COVID-19, the pandemic is associated with a significant economic burden. Although young people usually have a mild disease, they also suffer the consequences of quarantine and isolation.

Considering the far-reaching consequences of the COVID-19 pandemic among healthy young people and the still unknown long-term outcomes of the disease in this population, numerous investigators have started research on the risk factors for COVID-19. So far, comorbidities (obesity, Type 2 diabetes, atherosclerotic cardiovascular disease, and hypertension) and older age have been recognized as risk factors for death and hospitalization in the general population. However, the associations between the outcomes of COVID-19 and other demographic and clinical parameters in young adults have been addressed by a handful of studies [7,8]. Adequate understanding of the risk factors may help physicians and dieticians develop supportive strategies to protect people against infection with new variants of the virus in the future.

Literature data suggest that the morbidity of COVID-19 is associated with increased levels of inflammatory mediators [9], and that the severity of symptoms is linked with hyperactive immune response, due to increased levels of cytokines and chemokines [10]. The connection between diet and the immune system is well established, and available evidence shows that adequate nutrition is necessary for proper immune function. Poor diet weakens the immune defense system, which increases the susceptibility to disease [11]. Therefore, even though there currently seems to be no effective treatment for COVID-19, healthy eating habits, physical activity, rest, and not smoking contribute to improved immune function. Additionally, these behaviors decrease the likelihood of contracting COVID-19 and facilitate recovery in sick patients who have been infected [12]. Energy deficiency and malnutrition were reported among hospitalized patients with COVID-19. Nutritional assessment with the Modified Nutrition Risk in Critically Ill score, Global Leadership Initiative on Malnutrition criteria, or Nutritional Risk Score 2020 were shown to predict the outcome of patients with a critical illness due to COVID-19 [13,14,15,16,17]. The role of micronutrient intake in supporting the treatment was also described. Vitamin D deficiency was found in more than two-thirds of the admitted patients, and selenium deficiency, in 42% of the patients [1,18].

Another important issue is the relationship between intestinal dysbiosis and respiratory infections [19]. Some patients with COVID-19 were reported to have lower levels of gut bacteria, such as *Lactobacillus* and *Bifidobacterium* [20]. These and other bacteria affect the immune system both locally and systemically [21], and the modulation of gut microbiota is a promising strategy to improve immune mechanisms and thus reduce the effect of viral infections, including SARS-CoV-2 infection [22,23].

Recently, a link between the intake of omega-3 fatty acids, vitamin D, probiotics, multivitamin supplements, and a lower risk of positive test results for SARS-CoV-2 have been reported. However, the study was limited by the inclusion of women only [24]. Therefore, our population of healthy young adults included both women and men. This is very important, given the enormous burden of the pandemic on the healthcare system. Thus, the promotion of healthy diet and lifestyle ensuring gut microbiota eubiosis should be one of the main goals for researchers, particularly that an anti-inflammatory diet could be adjusted to prevent COVID-19 outcomes. In light of the above, the aim of this study was to describe the effects of eating behavior, gut microbiota, and physical activity on the risk of COVID-19 in a group of healthy, physically active young people.

## 2. Materials and Methods

### 2.1. Study Design and Sample

Between July 2020 and January 2021, a research project was conducted at the Department of Nutrition and Drug Research of Jagiellonian University Medical College (Cracow, Poland) as part of the MINIATURA 3 grant from the National Science Centre. The aim of the research was to assess physical activity, nutritional behavior, and gut microbiota of vegetarian and nonvegetarian men. Participants from Cracow were recruited through social media advertising and by snowball sampling. Passive recruitment via social media sites involved distributing recruitment materials (ads, posters, flyers) with the aim of attracting potential participants to contact the research team for more information and for consideration of enrollment.

As part of the study, participants were observed for a period of 1 week. Before enrollment, we made sure that it would be a usual week in the life of the participants, with similar diet and physical activity to the previous weeks. For individuals who had a special event scheduled during that time (e.g., wedding or birthday), observation would start a week after the event. During the training meeting, respondents were carefully instructed to behave as they usually do, to eat normally, and to perform a standard amount of physical activity. All participants were informed about study conditions and procedures and provided written consent to participate in the study. The study was conducted in accordance with the Declaration of Helsinki for medical research and with the positive approval of the Jagiellonian University Bioethics Commission (No. 1072.6120.5.2020 and 1072.6120.202.2019).

Participants received a Polar M430 watch to measure physical activity for a week. They were also instructed to keep dietary records for 7 consecutive days. After a week, they presented for a follow-up visit. During the visit, stool samples delivered by participants were collected for gut microbiota testing. Moreover, anthropometric and body composition measurements were performed, and a sociodemographic questionnaire was completed by participants.

The inclusion criteria were as follows: age 25–45 years, no chronic diseases, body mass index (BMI) in the range of 18.5–29.9 kg/m^2^, a vegetarian or traditional diet maintained for at least 1 year, and no antibiotic or probiotic therapy for at least 3 months before the measurements.

**Figure 1:**
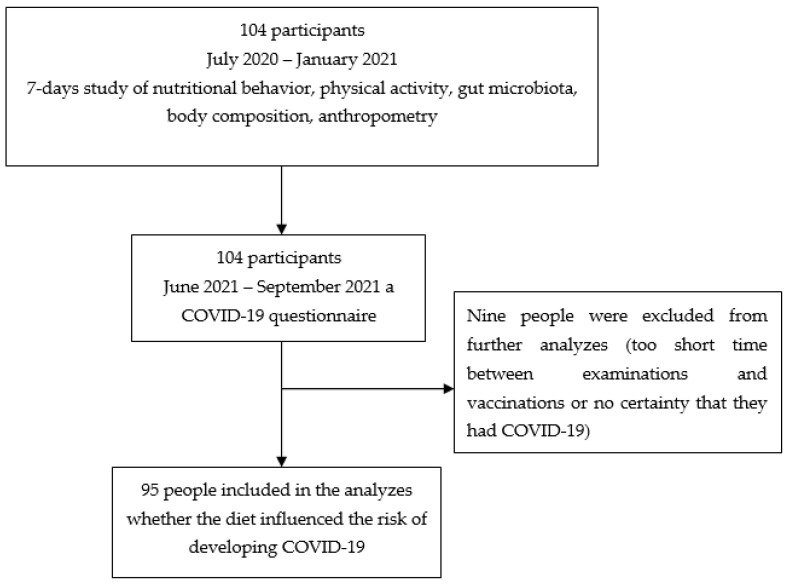
Selection of the study participants—own description.

**Figure 2:**
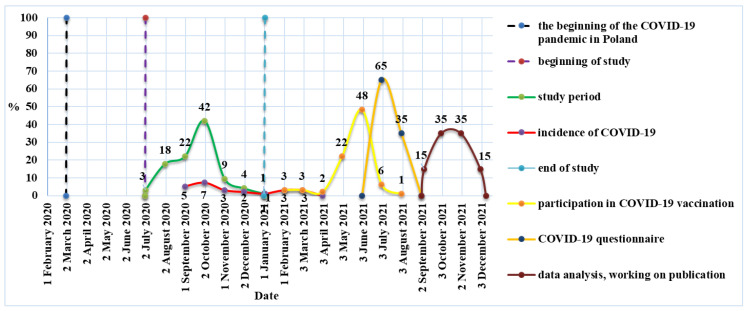
Timeframe for the course of the study—own description.

The study included 78 men: 41 on traditional diet and 37 vegetarians. An identical study including women was conducted in October and November 2020. The group included 17 women on traditional diet and 9 vegetarians (Figure 1). As there have been numerous reports on a milder course of COVID-19 in people on a plant-based diet, we decided to contact all study participants (104 people) in June 2021 and ask whether they had COVID-19 before or after the study, and thus we performed a follow-up analysis. If the answer was “yes” (based on positive polymerase chain reaction (PCR) or antibody test results or characteristic symptoms such the loss of smell and taste), participants were asked about the course and duration of the disease, the need for hospitalization, and vaccination against COVID-19 (including the date if vaccinated). Participation in this interview was voluntary and the questions were optional, but the response rate was 100%. Seven (29.2%) individuals fell ill before the study and 17 (70.8%) people after the study. These data were compared with physical activity, eating behavior, and gut microbiota at baseline. During the follow-up, all participants confirmed that they were still on a vegetarian or traditional diet. Nine participants were excluded because the time between the study and vaccination was too short or there was no certainty as to whether they had COVID-19. Therefore, the final study group included 95 people, of whom 24 had COVID-19. In eight persons, it was confirmed by positive PCR test results, and in seven persons, by positive antibody test results. Nine persons had typical COVID-19 symptoms, including loss of smell and taste. The study flowchart is presented in Figure 2.

### 2.2. Weight, Height, and BMI

Participants were informed about the test procedure, including the need to empty the bladder, if necessary, to minimize the risk of error, as well as to refrain from increased physical activity in the preceding 24 h. The height was measured with an accuracy of 0.1 cm using a Seca 213 portable stadiometer. The measurement was performed under standard conditions, barefoot and in an upright position. Body weight and body composition were measured by the electrical bioimpedance method (6.25 kHz, 50 kHz, 90 μA) using a calibrated segment analyzer (Tanita BC-418 MA, Tokyo, Japan) with an accuracy of 0.1 kg/0.1%. Participants were asked to remove their footwear and socks, and they had the skin on their feet cleaned. In accordance with the Tanita manual, the weighing platform was placed on the flattest possible surface and leveled to ensure accurate measurement. BMI was calculated as body weight (kg)/height (m^2^).

### 2.3. Physical Activity Recording

Participants received a Polar M430 watch for physical activity recording. The device was placed on the wrist of the nondominant hand. Participants were instructed to wear the device at all times, except when taking a shower or a bath, for full 7 days (24 h/day). They received written and oral instructions on how to wear the device. Polar M430 provided data on the total energy expenditure, number of steps, and sleep time.

### 2.4. Dietary Intake

Before the observation week began, participants received detailed instructions on how to prepare the dietary records for 7 consecutive days. They were shown examples, encouraged to use kitchen scales, or to refer to a website with the photos of products and their mass (http://www.ilewazy.pl, accessed from July 2020 to January 2021). Participants were carefully instructed to follow their standard diet during the observation week.

Upon receipt of food diaries from participants, data were entered into the Dieta 6.0 software (National Food and Nutrition Institute, Warsaw, Poland) in order to calculate the energy and nutritional value of daily food rations. The nutritional calculations included information on protein and individual amino acids, fat and individual fatty acids, carbohydrates including sucrose, fiber, cholesterol, minerals, and vitamins. The values of macronutrients and micronutrients were referred to the Polish norm according to the age and sex of the participants [25]. To evaluate the prevalence of nutrient adequacy, adequate intake (AI) for water, recommended dietary allowance (RDA) for protein, and recommended dietary allowance (RDA) and AI were used for micronutrients, vitamins, fatty acids, and dietary fiber. Based on nutritional recommendations, participants were divided into three groups: average daily consumption of >500 g of fruit and vegetables and >10 g of nuts, failure to meet one of the conditions, failure to meet any of the conditions.

### 2.5. Dietary Inflammatory Index (DII)

The Dietary Inflammatory Index was calculated based on the methods proposed by Shivappa et al. [26]. A regionally representative database harmonizing eleven nutrition surveys and reflecting the diets of diverse populations was used to assess basic statistics for each out of 45 food items. In the examined sample, an individual’s food-specific dietary intake was linked to these “standard global parameters” and the Z scores for each food product were obtained by subtracting the standard mean from the amount of the reported consumption and next by dividing by standard deviation. To improve distribution of such right-skewed data, the conversion to percentile scores was applied, and, further, it was centered on 0 by doubling and then subtracting “1”. The multiplication by the corresponding overall inflammatory effect score assessed from the global composite dataset allowed us to obtain the food parameter-specific DII score. Finally, summation of them resulted in creation of the overall DII score. In the original article, the 45 food items were included in calculation; however, we had evaluated only 30, namely, onion, garlic, coffee, tea, alcohol, energy, protein, carbohydrates, fiber, vitamins C, D, A, E, B6, B12, B1, B2, and B3, folate, β-carotene, saturated fatty acids, total fat, monounsaturated fatty acids, polyunsaturated fatty acids, n-3 fatty acids, n-6 fatty acids, cholesterol, magnesium, iron, and zinc.

### 2.6. Analysis of the Gut Microbiota

The microbiological assessment of stool samples included the analysis of the presence and number of selected types of bacteria (KyberKompactPro test, Poland). The test is based on microbiological culture performed from several dilutions of a stool sample and on real-time PCR assays. Quantitative results for bacteria were expressed as cfu/g (colony forming units per gram of stool). The stool samples were immediately sent directly to a microbiological laboratory in Poznań (Poland). No more than 36 h elapsed between stool sample collection and its delivery to the laboratory. Bacteria of the species *Faecalibacterium prausnitzii* and *Akkermansia muciniphila* were quantified by PCR method. DNA for determination was isolated from 180–220 mg of feces. The QIAamp Fast Stool Mini Kit DNA kit (QIAGEN, Valencia, CA, United States) was used for DNA isolation. The assay was performed using the 7300 Real-Time PCR System, from Applied Biosystem. For *Clostridioides difficile*, Toxin A/B—a fecal molecular assay by Real-Time PCR was used (RIDA^®^GENE CD Toxin A/B, R-Biopharm, Darmstadt, Germany). For *Escherichia coli, Escherichia coli Biovare, Proteus* spp., *Providencia* spp., *Morganella* spp., *Pseudomonas* spp., *Klebsiella* spp., *Enterobacter* spp., *Citrobacter* spp., *Serratia* spp., *Hafnia alvei, Enterococcus* spp., *Bifidobacterium* spp., *Bacteroides* spp., *Lactobacillus* spp., *H_2_O_2_*—*Lactobacillus, Clostridium* spp. tests were performed by culture method. The detailed results of the gut microbiota analysis are published elsewhere [27,28].

### 2.7. Power Analysis

Post hoc, we calculated an achieved power of the multiple logistic regression that tests the main hypothesis that two groups of people consuming or not consuming recommended amounts of fruits and vegetables (FV ≥ 500 g and nuts ≥ 10 g) differ significantly in the risk prevalence of COVID-19. In the light of our results, we incorporated the following data: total sample size: 95 participants; odds ratio of having contracted COVID-19 for comparison of two groups: 5.93; probability of having contracted of COVID-19 when inadequate amounts of fruits and vegetables are consumed: 0.36; amount of variability in the main predictor that is accounted for by covariates: 0.25; and probability of consuming recommended amounts of fruits and vegetables 0.42. Additionally, as a gold standard, two-tailed test was considered, and probability of type I error at a level 0.05 was assumed. Under the above constraints, we obtained highly satisfactory power of multiple logistic regression equal to 0.93.

### 2.8. Statistical Analysis

Descriptive statistics were calculated: mean, standard deviation, median, and the first and third quartiles (Q1–Q3). Compliance with the normal distribution of quantitative variables was checked using the Shapiro–Wilk test. In order to check the differences between the two groups (participants who have not contracted and have contracted COVID-19) for the analyzed quantitative or ordinal variables, the Student’s *t*-test or the Mann–Whitney U-test was used. The Kruskal–Wallis analysis of variance was used to check the differences between the three groups (depending on the average daily the intake of vegetables, fruits, and nuts). The multivariable logistic regression was applied to calculate odds ratios (ORs) and 95% confidence intervals (CIs). Statistical analyses were performed using PS IMAGO PRO 7 (IBM SPSS Statistics 27, Armonk, NY, USA), STATISTICA 13.3 (TIBCO Software Inc., Palo Alto, CA, USA), and R 4.04 (Development Core Team, Vienna, Austria) softwares. The level of statistical significance was set at *p* < 0.05.

## 3. Results

### 3.1. Characteristics of Participants

The study included 95 young adults, including 73 men (76.8%) and 22 women (23.2%). The mean age of participants was 34.66 ± 5.76 years, and individuals who contracted COVID-19 were older than those who did not (*p* = 0.0212). The descriptive characteristics of the study group are presented in Table 1.

**Figure 3:**
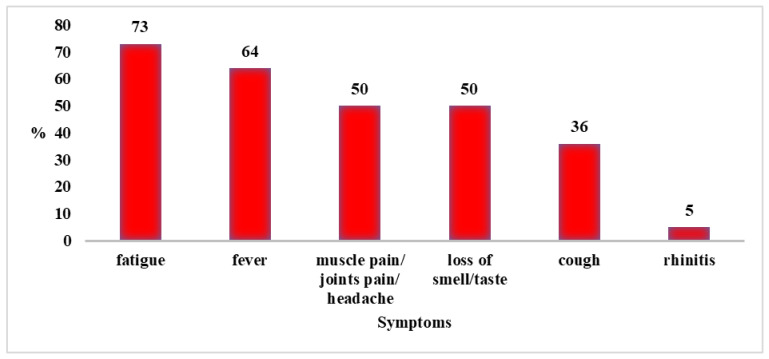
Disease symptoms in people who contracted COVID-19 (*N* = 24).

Of the 95 people, 24 became ill with COVID-19 (24.3%). The median duration of the disease was 5 days (Q1–Q3: 3–7). None of the patients required hospitalization. Disease symptoms among study participants are shown in Figure 3.

### 3.2. Findings

Details of dietary intake in the study population along with a comparison between adults who contracted COVID-19 versus those who did not are presented in Table 2. The intake of energy, water, plant protein, carbohydrates, and dietary fiber was significantly lower in individuals who reported COVID-19 than in those who did not. The mean intake of energy was 2120 kcal in the COVID-19 group versus 2274 kcal in people who did not report COVID-19, while dietary fiber intake was 18.8 g and 26.8 g, respectively.

In addition, there were significant differences in the minerals and vitamins intake. People with a history of COVID-19 showed a lower dietary intake of potassium, magnesium, iron, zinc, copper, manganese, vitamin E, thiamine, vitamin B6, and folates than those without a history of the disease (*p* < 0.05). In addition, the diets of participants who did not develop COVID-19 had negative DII scores, indicating the anti-inflammatory properties of their diet, in comparison with participants who contracted COVID-19 (−1.08 vs. 0.98).

We assessed compliance with dietary recommendations in terms of the percentage of reference intake for individual nutrients, vitamins, and minerals (Table 3). People without a history of COVID-19 showed overall good compliance with dietary recommendations for most substances. On the other hand, individuals who contracted the disease showed an insufficient percentage of reference intake. Specifically, significant differences between groups were found for the intake of water, carbohydrates, potassium, magnesium, iron, zinc, copper, manganese, vitamin E, thiamine, vitamin B6, dietary fiber, and folates (*p* < 0.05).

Data on the consumption of selected food products by respondents are presented in Table 4. The participants of our study who did not contract COVID-19 showed a higher daily consumption of groats and rice, nuts, fruit and vegetables, and legumes, compared with those who reported a history of the disease. In addition, we assessed the daily consumption and portions of selected products. The groups differed in the consumption of garlic and nuts (Table 4). Among young adults with a history of COVID-19, only 4.2% consumed more than 1 g of garlic per day, compared with 22.5% of individuals who did not contract COVID-19. Similarly, a significantly lower percentage of young adults with a history of COVID-19 consumed more than 10 g of nuts per day when compared with individuals without a history of COVID-19 (33.3% vs. 67.6%; Table 4).

#### 3.2.1. Analysis of the Gut Microbiota

**Figure 4:**
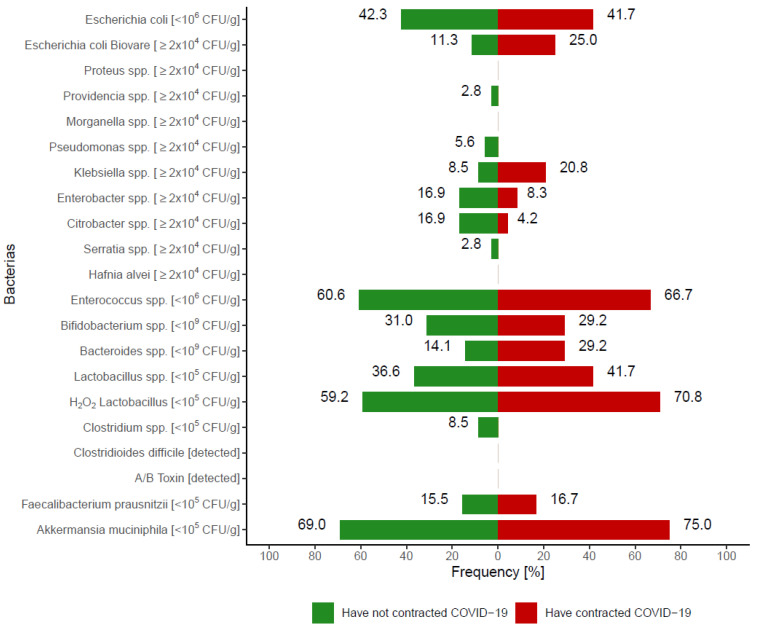
The percentage of people with a number of individual bacteria outside reference values (*p* > 0.05).

The percentage of people with a number of individual bacteria outside reference values is shown in Figure 4. The Y axis shows the cut-off points for the number of individual bacteria, indicating the number of bacteria outside the reference values. There were no significant differences between participants who contracted COVID-19 versus those who did not (*p* > 0.05). The greatest disparity between groups was shown for *Klebsiella* spp., which was more often present in people with a history of COVID-19 versus those without. However, the difference was not significant (*p* = 0.1012).

#### 3.2.2. Nutritional Behavior

**Figure 5:**
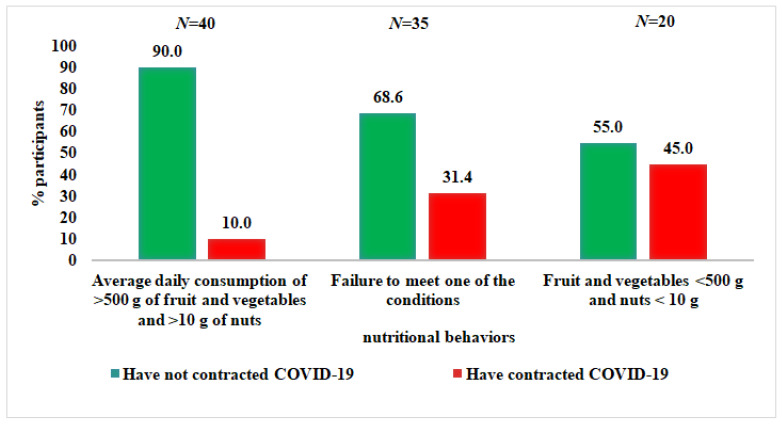
Associations between the consumption of vegetables, fruits, and nuts and the incidence of COVID-19.

In the group of people with an average daily consumption of more than 500 g of vegetables and fruit and of more than 10 g of nuts, only 10% of people fell ill with COVID-19, compared with 45% of those who did not meet any of the dietary requirements (*p* = 0.0076) (Figure 5).

The OR for the association between consumption of selected food products and the risk of COVID-19 was calculated (Table 5). In the crude model, the risk of contracting COVID-19 in individuals who consumed insufficient amounts of fruit and vegetables as well as nuts was more than 7-fold higher than in those with an adequate consumption of these products. In the fully adjusted model (after adjustment for the type of diet, sex, marital status, age, body fat percentage, smoking, and the presence of *Klebsiella* spp. in fecal samples), the odds were more than 12-fold higher than in people with high consumption of both fruit and vegetables and nuts, independently of the abovementioned predictors (OR = 12.22, 95% CI: 2.47–78.12). Similarly, individuals with an insufficient intake of a single food product showed an almost 6-fold higher risk of COVID-19 (OR = 5.73, 95% CI: 1.49–27.82). The results were similar after adjustment for all the variables used in model 3, except *Klebsiella* spp., which confirms the robustness of the findings (Table 5).

**Figure 6:**
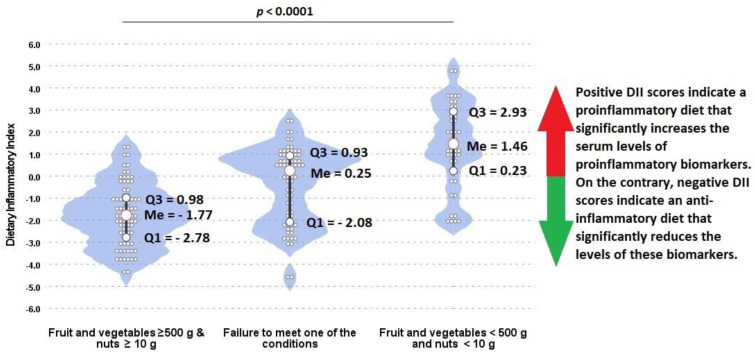
Associations between the consumption of vegetables, fruits, and nuts and Dietary Inflammatory Index among participants. Wider areas on violin plots refer to higher probability of occurrence. Q1, Q3, Me: quartiles and median of distribution.

Detailed data on the consumption of selected food products depending on the consumption of vegetables, fruit, and nuts (both in grams and by dietary thresholds) are presented in Table 6. There were significant differences between groups in the consumption of seeds, nuts, vegetables, fruit, and legumes (*p* < 0.05). In the group of people with an average daily consumption of more than 500 g of vegetables and fruit and over 10 g of nuts, there were more vegetarians compared with the group of people who did not meet any of these dietary recommendations (55.0% vs. 20.0%). Moreover, participants with balanced diet including the average daily consumption of >500 g of fruit and vegetables and >10 g of nuts had a negative DII scores, indicating the anti-inflammatory properties of their diet, in comparison with participants whose diet was not well balanced and contained few vegetables, fruits, and nuts (−1.77 vs. 1.46) (Figure 6).

Example menus of people who contracted COVID-19 and those who did not are included with permission in Supplementary Materials (Tables S1 and S2). There was a noticeable difference in terms of vegetable and fruit intake, as well as the variety of foods consumed between groups.

## 4. Discussion

Our results confirm that nutrition is a particularly important issue in the prevention of COVID-19, as stated by some clinicians and nutritionists since the onset of the pandemic. Beginning in spring 2020, medical experts, including Dr Zbigniew Martyka and Professor Piotr Kuna, have underlined the importance of optimal immune function (achieved via proper nutrition and physical activity) in the context of reducing the risk of SARS-CoV-2 infection and COVID-19 [29,30,31]. Around that time, in March 2020, a paper by Professor Philip Calder was published on nutrition, immunity, and COVID-19, in which he summarized the current knowledge on immunomodulation and provided nutritional recommendations for reducing the risk of COVID-19 [32]. The paper discusses in detail the individual nutrients and their role in shaping innate and acquired immunity as well as indicates good dietary sources of these ingredients (including vegetables, fruits, nuts, legume seeds, meat, fish, poultry, and dairy products).

In April 2021, the Committee on Human Nutrition Science of the Polish Academy of Sciences issued a statement on dietary recommendations during the COVID-19 pandemic. The statement reiterated that numerous dietary components are involved in the regulation of the immune system, and that vitamins D, C, A (including beta-carotene), E, B_6_, B_12_, folic acid, zinc, copper, selenium, iron, amino acids, and n-3 and n-6 polyunsaturated fatty acids, as well as gut microbiota, are important for various defense processes. Moreover, it recommended to eat a varied diet, with a large proportion of plant-based food and a proper amount of animal-based food according to age. However, these recommendations appeared more than a year after the pandemic was declared in Poland, and they were not promoted by the Polish Ministry of Health [33].

### 4.1. Study Group

Research conducted by The National Institute of Public Health in 2019 in Poland revealed that in the group of men aged 18–40 years, 45.5% had a normal body weight, 44.5% were overweight, and 9.7% were obese, while in the group aged 41–64 years, the respective values were 23.0%, 57.4%, and 19.7% [34]. In addition, according to the data of Statistics Poland, only 55% of adults ate fruit every day in 2019, the least frequent people aged 20–29 (47%). Daily consumption of fruit and vegetables decreased in comparison with the year 2014 by 4 and 5 percentage points, respectively. A reduction in such frequent consumption of fruit was particularly noticeable among twenty-year-olds (about 6–7 percentage points). In the case of daily consumption of vegetables, the greatest decline was observed among 40 year-olds (6 percentage points). Moreover, only 51% of adults ate vegetables every day [35]. In the study group, 68.1% of the respondents consumed fruit every day and 87.3%—vegetables. According to the Public Opinion Research Center in 2019, about 34% of men and about 19% of women in the group aged 25–45 years smoked cigarettes in Poland, with only about 13% in the study group [36].

Our study group included healthy young nonobese and physically active people, nonsmokers, with higher education, who followed a traditional or vegetarian diet and mostly consumed vegetables every day. Therefore, they differed significantly from the general population of this age in Poland. The results were presented and then conclusions were drawn for such a specific group of respondents.

### 4.2. Nutritional Behaviors

The results of the current study prove that proper nutritional behavior is an important nonpharmacological measure to reduce risk of the spread of SARS-CoV-2 infection and COVID-19. To our knowledge, this is the first study conducted in Poland among healthy, young, nonobese adults during the COVID-19 pandemic that assessed the impact of nutritional behavior and gut microbiota on the risk of COVID-19 contraction.

The COVID-19 pandemic has necessitated the development of disease prevention measures and treatment methods in a short time. Despite intensive research, few effective early-stage preventive and therapeutic interventions for COVID-19 are currently available [37]. Micronutrient deficiencies, especially of vitamins A, B, C, and D, as well as minerals such as zinc, iron, and selenium, are common among patients with COVID-19 and may increase the risk of death [38].

According to the Nationwide Seroepidemiological Study COVID-19: OBSER-CO, conducted in Poland from 29 March to 14 May 2021, 29.5% of people aged 20–39 years contracted COVID-19 [39]. This is in line with the results of the current study, which showed that 24.5% of respondents had contracted COVID-19 by summer 2021.

### 4.3. Macronutrients

This study showed that the nutrition of people with a history of COVID-19 was characterized by a significantly lower energy value, water content, and intake of plant protein, carbohydrates, and fiber, compared with people without a history of COVID-19. The consumption of a certain amount of protein with high biological value is crucial for the optimal production of antibodies [16]. Dietary protein levels below the recommended level of 0.8 g/kg body weight are a well-known risk factor for infection [40,41]. However, protein derived from foods such as processed meat and cheese may have pathological influences on postprandial lipoprotein/glucose/insulin metabolism, promoting lipogenesis and increasing inflammation [42,43]. On the other hand, the anti-inflammatory properties of plant-based proteins are well recognized [44]. In our study, people who did not develop COVID-19 showed a higher dietary intake of plant-based protein than those with COVID-19.

In our study, people who contracted COVID-19 revealed a lower arginine intake than individuals with no COVID-19 history, but the difference was of borderline significance. Similar results were obtained by Rees et al. [45], who showed that 53.1% of people who contracted COVID-19 had arginine concentrations in blood below 50 μmol/L, compared with 3.6% of the control group. Arginine (L-arginine) is an amino acid involved in numerous biological processes, including the host immune response [46]. Recently, amino acid metabolism has been shown to be a key factor in the pathophysiology of COVID-19 [28]. Specifically, in patients with COVID-19 (especially the most severe cases), reduced plasma L-arginine levels and enhanced arginase activity were reported [47,48].

In our study, people who contracted COVID-19 had a significantly lower intake of dietary fiber: 18.8 g versus 26.8 g in individuals without COVID-19. This is in line with the study of Ponzo et al. [49] on healthcare professionals, which showed that people who contracted COVID-19 supplied less fiber to their diet than those who did not develop the disease (20.6 g vs. 23.2 g).

In a study by Park et al. [50], for every 10 g increase in dietary fiber per day, the relative risk of death from infectious and respiratory diseases decreased by 34% and 18%, respectively, in men, and by 39% and 34%, respectively, in women. In another observational study involving 11,897 men [51], dietary fiber consumption was associated with a reduced risk of chronic obstructive pulmonary disease (not necessarily related to infection). Dietary fiber is also necessary to maintain the proper function of the beneficial gut microbiota [13]. As a result of bacterial activity in the intestine, numerous metabolites are produced, including short-chain fatty acids exerting anti-inflammatory, immunomodulatory, and gene expression effects through DNA methylation [52,53].

### 4.4. Gut Microbiota

Dietary fiber was also reported to increase the diversity of the gut microbiota and promote health-related bacteria such as *Bifidobacterium* spp. and *Lactobacillus* spp. [54], which may contribute to limiting the growth of harmful pathogens, including *Clostridium* spp. [55]. Intestinal microflora rich in *Bifidobacterium* spp., *Faecalibacterium* spp., *Ruminococcus* spp., and *Prevotella* spp. is associated with lower systemic inflammation, characterized by decreased levels of high-sensitivity C-reactive protein and interleukin IL-6 [56]. In the current study, no significant differences in the occurrence of any type of the bacteria were shown between people who contracted COVID-19 and those who did not; however, there was a tendency for a higher prevalence of *Klebsiella* spp. in the COVID-19 group.

### 4.5. Micronutrients

While the beneficial role of any single nutrient in the fight against COVID-19 has not been clearly established, the importance of nutrition for optimal immune function is well known [57]. Alfano et al. [58] reported hypokalemia to be a common electrolyte disturbance among patients hospitalized for COVID-19 (41% of cases). In a study by Chen et al. [59], hypokalemia was present in 34 of the 40 seriously and critically ill patients (85%). Hypokalemia is one of the disorders that may reflect the progression of COVID-19 and thus should be closely monitored. Given these risks, potassium levels should be maintained above 4 mmol/L in patients with COVID-19 and hypokalemia [60,61]. Low potassium content caused by an insufficient dietary intake may exacerbate the course of COVID-19 because oral potassium administration increases its blood concentrations [62]. In our study, people who did not contract COVID-19 had higher potassium content in their diet than those who contracted the disease. They also showed an adequate percentage of reference intake for potassium: 107.4% versus 89% in people reporting a history of COVID-19.

Individuals who contracted COVID-19 showed a lower dietary intake of magnesium than people without COVID-19. Magnesium is involved in over 600 enzymatic reactions in the body, including those that contribute to enhanced immune and inflammatory responses shown by COVID-19 patients [63]. In some respects, the pathogenesis of COVID-19 resembles processes observed in magnesium deficiency, such as reduced T-cell count, elevated plasma levels of inflammatory cytokines, and endothelial dysfunction. Therefore, it was hypothesized that low magnesium status, which is quite common, may contribute to the progression from mild to critical clinical symptoms of COVID-19 [64].

Our study also revealed a significantly lower supply of iron and zinc to the diet in people who contracted COVID-19 versus those who did not. Patients with COVID-19 tend to have reduced hemoglobin levels (indicating the presence of anemia) and abnormally high ferritin levels. A study of 67 COVID-19 patients from Singapore showed that patients staying in the intensive care unit had a significant gradual reduction in hemoglobin levels, compared with patients who were not admitted to the intensive care unit [65]. In a report of 5700 patients hospitalized for COVID-19 in the New York City area, ferritin levels were pathologically high [66], which is consistent with the result of a previous study including 113 Chinese patients who died from coronavirus [67].

Zinc deficiency is another major factor underlying the dysfunction of the immune system in selected human populations [68]. Zinc is considered a prophylactic or adjunct therapy for COVID-19, and clinical trials highlighting the importance of this trace element in the global pandemic are underway [69]. Another significant element is copper. Its antibacterial and antiviral activity is well understood [70]. Copper ions exert protective effects against viruses, including coronaviruses [71,72]. Copper deficiency can cause neutropenia and immunosuppression through decreased proliferation of T cells [73]. Raha et al. [72] hypothesized that the enrichment of plasma copper levels would increase both innate and adaptive immunity in humans. Moreover, owing to its strong antiviral activity, copper can act as a preventive and therapeutic agent against COVID-19. Considering that dietary copper deficiency affects both innate and adaptive immunity [74] and that people with copper deficiency show considerable susceptibility to infections [72], it is not surprising that our study revealed low copper consumption among people infected with COVID-19.

### 4.6. Vitamins

Poor nutritional status is associated with inflammation and oxidative stress, which in turn can affect the immune system. Dietary components with particularly high anti-inflammatory and antioxidant capacity include vitamins, such as vitamin C and vitamin E, and carotenoids [13]. Vitamin B also plays a role in the prevention of COVID-19 [13]. In our study, the diets of respondents who contracted COVID-19 were characterized by a lower intake of vitamin E, thiamine, and folate, compared with the diets of people who did not suffer from COVID-19. A study including 71 adults showed an increased expression of various genes related to the immune response as a result of tocopherol or tocotrienol supplementation [75]. A beneficial effect of vitamin E supplementation in elderly people with pneumonia was demonstrated. It was associated with a reduction in the rehospitalization rate by 63% [76]. In a study on pregnant women with COVID-19, lower levels of vitamin E were claimed to support increased oxidative stress in the etiopathogenesis of COVID-19 and to be associated with complex adverse perinatal outcomes [77].

In our study, only 33.3% participants who contracted COVID-19 consumed more than 10 g nuts per day, in contrast to 67.6% in the group who did not contract the disease. Nuts are not only an excellent source of vitamin E and B but also of zinc, selenium, magnesium, copper, and arginine, which may account for their protective potential in COVID-19 [78]. Although nuts are energy-dense, nut consumption was reported to have beneficial effects attributed to fatty acid, vegetable protein, fiber, vitamin, mineral, carotenoid, and phytosterol content with potential antioxidant activity [79]. Nuts also contain phenolic compounds that show immunoprotective effects, especially through antioxidant and anti-inflammatory properties, in adults [80].

Thiamine deficiency in COVID-19 may lead to insufficient elimination of the virus, cause an insufficient antibody response, and aggravate symptoms [81]. Although the role of thiamine in patients with COVID-19 is unclear, Vatsalaya et al. [82] showed that a 3-week therapy with thiamine at a dose of 200 mg per day significantly decreased the baseline level of IL-17 and, at the same time, increased the level of IL-22 (anti-inflammatory response) in 16 patients with COVID-19 (proinflammatory origin due to heavy drinking). Adjunctive therapy with thiamine in critically ill patients with COVID-19 was also described by Al Sulaiman et al. [83], who reported that thiamine use was significantly associated with in-hospital and 30-day mortality as well as a lower incidence of thrombosis.

In addition, vitamin B_9_ (folate) was suggested as a possible beneficial factor in the prevention of SARS-CoV-2 infection, through its involvement in innate immunity and, in particular, through natural killer cell cytotoxicity, which may reduce the risk of infection [84].

### 4.7. Dietary Intake and Dietary Inflammatory Index

It was shown that among people whose balanced diet was rich in vegetables, fruits, nuts, and legumes, only 10% contracted COVID-19, compared with 45% of those whose diet was not balanced and was poor in these products. Subjects with an average daily consumption of less than 500 g of fruit and vegetables and less than 10 g of nuts had a 7.36-fold higher risk of developing COVID-19 compared with those who consumed more than 500 g of fruit and vegetables and more than 10 g of nuts. Similar odds for developing COVID-19 were reported by Moludi et al. [85], who performed a diet analysis based on the Dietary Inflammatory Index (DII). Study participants with a maximum proinflammatory-energy-adjusted E-DII score had a 7.26-fold higher risk of COVID-19, compared with those in tertile 1 (E-DII T3 vs. E-DII T1: OR = 7.26; 95% CI: 2.64–9.94). Our results show that participants with balanced diet including average daily consumption of >500 g of fruit and vegetables and >10 g of nuts have very low DII scores, indicating the anti-inflammatory properties of their diet in contrast to participants whose diets were not well balanced and contained few vegetables, fruits, and nuts (−2.31 vs. 0.84). Moreover, R. Perez-Araluce et al. showed that that higher adherence to the Mediterranean diet may be associated with a lower subsequent risk of COVID-19. They noticed significant results in the risk reduction by more than 60% for the categories of higher adherence to the Mediterranean diet. However, these results applied only to individuals who were not health professionals [86].

Fruit and vegetables are known to provide compounds that are highly relevant for proper immune function, and also other bioactive compounds, such as polyphenols [82]. In our study, participants who contracted COVID-19 consumed lower amounts of vegetables and fruit. This is in line with the results of the Nutri-Net-Santé cohort study by Deschasaux-Tanguy et al. [87], including 7766 participants. People who contracted COVID-19 consumed 410 ± 200 g of vegetables and fruit daily, compared with 460 ± 200 g in the group that did not contract the disease (*p* < 0.0001). Our results regarding vegetable consumption are also consistent with a recent prospective study published in the UK Biobank database [88]. Garlic (*Allium sativum* L.) is a common functional food and has potential antiviral activity, especially by its organosulfur compounds’ (alliin and allicin) immunomodulating effects [89,90]. Mösbauer et al. studied the effect of allicin on SARS-CoV-2-infected Vero E6 and Calu-3 cells and demonstrated the immunomodulatory activity and antiviral properties of biocompatible doses of allicin in SARS-CoV-2- 2-infected cell cultures [91]. In our study, we found that people who have not contracted COVID-19 consumed more garlic; thus, it supports the in vitro findings.

### 4.8. The Role of Healthy Lifestyle in Prevention of COVID-19

We can hypothesize that if the above results translated to the entire population of young people without comorbidities, it could be expected that the number of people contracting COVID-19 would decrease significantly, which would provide health, economic, social, and psychological benefits. Importantly, our results may have a wider application than the SARS-CoV-2 setting, as the immune system is constantly exposed to various other types of pathogens. We currently do not know when medicine will deal with SARS-CoV-2 infection. Moreover, other viruses may cause new epidemic outbreaks in the future. Therefore, it is important to emphasize the role of proper nutrition for optimal immune function. Physical activity and a proper diet ensuring the supply of all necessary nutrients should form the basis of health maintenance. This applies even to cases where there is access to vaccinations or effective pharmacotherapy, given that vaccinated people are still at risk of SARS-CoV-2 infection and the development of COVID-19.

There are numerous risk factors for COVID-19, including both nonmodifiable factors such as age and genetic conditions, as well as factors that can be modified, including nutrition, physical activity, comorbidities (e.g., obesity), gut microbiota composition, smoking, amount of sleep, stress, and exposure to air pollution. Among the modifiable risk factors, the change of diet is relatively easy to introduce, and a simple modification can bring tangible health benefits. Proper nutrition is important not only for disease prevention, the consumption of vegetables, fruit, nuts, adequate fluid intake, and vitamin D supplementation are also indispensable during illness to ensure an adequate supply of water, protein, magnesium, zinc, vitamins C, D, and E, fiber, and beta-carotene indispensable for optimal immune function.

Our results support the findings of other researchers that the immunomodulatory impact of some bioactive components, especially vitamins A, C, D, and E, the minerals zinc and selenium, and essential fatty acids have roles in reducing the risk and severity of COVID-19 infection by supporting innate and adaptive immunity [92].

### 4.9. Healthy Lifestyle Recommendation

Based on the menus of participants who did not become ill and a review of the literature regarding dietary recommendations to reduce risk of contracting COVID-19, we recommend moderate daily physical activity, maintenance of normal body weight, and consumption of the appropriate amounts of red and green peppers, broccoli, onions, garlic, carrots, tomatoes, cauliflower, sauerkraut, oranges, grapefruits, apples, plums, buckwheat groats, oatmeal, whole meal bread, yoghurt, curdled milk, nuts, seeds, pulses, ginger, fish, lean meat, poultry, eggs, olive and rapeseed oil, bee products, drinking mineral water, green tea, as well as cod liver oil as a source of vitamin D, and omega 3 fatty acids. [32,93,94,95,96,97].

### 4.10. Strengths and Limitations

In this study, we assessed the effect of nutritional behavior, physical activity, and gut microbiota on the incidence of COVID-19 among healthy, nonobese people from Cracow between March 2020 and April 2021. We included a homogeneous group of people and analyzed reliable data obtained from food diaries, which constitutes the strength of the study. Moreover, the risk of infection can be also influenced by air pollution, so by examining individuals from a single city, we were able to exclude this factor as a potential confounder. However, there are also potential limitations that need to be acknowledged. First, the sample size was relatively small. However, considering the restrictions and the duration of the pandemic, the number of participants seem to be sufficient. Second, at the time of the study, there was mainly the Alpha variant of coronavirus, and it is difficult to say how the results would translate to the current setting with the dominant Delta variant, and possibly Omicron in the near future. Third, gut microbiota was not determined by next-generation sequencing. Fourth the history of COVID-19 was determined based on the declarations of participants, who confirmed the infection using the PCR or antibody test. Fifth, we assumed that the diet and gut microbiota of the participants and their physical activity did not change over time. Sixth, we assumed that all participants had similar probability of contact with persons ill with COVID-19 from March 2020 to April 2021. Seventh, we are aware that the risk of coronavirus infection may also be affected by current levels of stress, overwork, and genetic differences that affect immune function. However, as we were not able to test for these factors, we assumed that they were similar among participants. Seventh, vitamin D levels have not been studied with biochemical tests, and its main source is endogenous synthesis, therefore, based on dietary data alone, conclusions and correlations cannot be drawn in this case. In addition, results are limited to healthy young nonobese and physically active people. Finally, we did not measure other exposures of the participants, such as how rigorous they were in applying preventive measures (e.g., hand washing, masks, physical distancing, etc.).

## 5. Conclusions

A proper diet rich in vegetables and fruit (including adequate amounts of garlic), as well as nuts, wholegrain cereal products, and legumes, covers the need for vitamins, minerals, and other bioactive substances. As such, it can be an effective measure in reducing the risk of COVID-19 in healthy physically active young people without obesity and with optimal immune function. Our study revealed no differences in physical activity levels or gut microbiota composition between people who contracted COVID-19 and those who did not. However, differences in diet were shown. People with a history of COVID-19 consumed lower amounts of groats and rice, fruit and vegetables (including garlic), nuts, and legumes. They had a lower dietary intake of water, plant protein, dietary fiber, potassium, magnesium, iron, zinc, copper, vitamin E, thiamin, vitamin B_6_, and folates than that of people who did not contract COVID-19. Young, healthy, nonobese, moderately physically active people who ate a balanced diet with an average daily consumption of more than 500 g of vegetables and fruit and over 10 g of nuts may have an 86% lower risk of developing COVID-19 compared with those whose diet was not balanced and who consumed fewer vegetables, fruits, and nuts. It is well recognized that proper nutrition, physical activity, and maintenance of normal weight ensure optimal function of the immune system and are thus necessary to maintain health. Therefore, their importance as a preventive measure cannot be marginalized. As nutritionists, dietitians, and medics, we call for immediate social campaigns educating the public on how to eat properly and maintain normal weight to ensure optimal immune function, especially in the context of SARS-CoV-2 infection and COVID-19.

A healthier population might be able to counteract SARS-CoV-2 virus, preventing new waves of infections. Additionally, a well-balanced diet may have a protective influence against other viruses, such as the influenza virus.

## Figures and Tables

**Table 1:** Anthropometric and sociodemographic characteristics of the study participants.

Variable		Total *N* = 95		Have Not Contracted COVID-19 *N* = 71		Have Contracted COVID-19 *N* = 24		
		$\bar{x}\pm \mathrm{SD}$		$\bar{x}\pm \mathrm{SD}$		$\bar{x}\pm \mathrm{SD}$		Student’s *t*-test *p*
Age (years)		34.66 ± 5.76		33.90 ± 5.72		36.92 ± 5.40		0.0212
Body weight (kg)		73.39 ± 12.27		73.81 ± 12.31		72.17 ± 12.36		0.6921
Height (cm)		176.97 ± 8.90		177.74 ± 8.88		174.71 ± 8.75		0.2202
BMI (kg/m2)		23.32 ± 2.76		23.26 ± 2.82		23.51 ± 2.63		0.6431
TEE (kcal)		2547 ± 445		2581 ± 459		2445 ± 393		0.3167
PAL		1.50 ± 0.17		1.50 ± 0.18		1.47 ± 0.12		0.5443
Sleep duration (h)		7:22 ± 0:49		7:26 ± 0:50		7:10 ± 0:45		0.1348
Steps		12,080 ± 4526		12,226 ± 4768		11,648 ± 3778		0.6067
**Variable**	**Category**	*N*	%	*N*	%	*N*	%	**Chi^2^** ***p***
Sex	Men	73	76.8	55	77.5	18	75.0	0.8046
	Women	22	23.2	16	22.5	6	25.0	
Marital status	Single/divorced	48	50.5	39	54.9	9	37.5	0.1398
	Married/cohabiting	47	49.5	32	45.1	15	62.5	
Diet	Traditional	54	56.8	38	53.5	16	66.7	0.2610
	Vegetarian	41	43.2	33	46.5	8	33.3	
BMI (kg/m^2^)	Normal body weight	64	67.4	48	67.6	16	66.7	0.9324
	Overweight	31	32.6	23	32.4	8	33.3	
BF (%)	Under fat	9	9.6	8	11.4	1	4.2	0.5676
	Normal	69	73.4	50	71.4	19	79.2	
	Overfat	16	17.0	12	17.1	4	16.7	
Smoking	No	83	87.4	61	85.9	22	91.7	0.5009
	Yes	12	12.6	10	14.1	2	8.3	
Education	Secondary	7	7.4	7	9.9	0	0.0	0.2393
	Higher	88	92.6	64	90.1	24	90.0	
How do you rate your physical activity in your free time?	Low	19	20.0	16	22.5	3	12.5	0.4448
	Moderate	52	54.7	36	50.7	16	66.7	
	High	24	25.3	19	26.8	5	20.8	
Vitamin supplementation	I don’t use	30	31.6	23	32.4	7	29.2	0.8841
	Periodically	21	22.1	15	21.1	6	25.0	
	Regular	44	46.3	33	46.5	11	45.8	
Supplementation with minerals	I don’t use	50	55.6	38	56.7	12	52.2	0.8236
	Periodically	23	25.6	16	23.9	7	30.4	
	Regular	17	18.9	13	19.4	4	17.4	

TEE—total energy expenditure; BMI—body mass index; BF—body fat; PAL—physical activity level; *N*—number of participants; $\bar{x}$ —arithmetic mean; SD—standard deviation. Bold values denote statistical significance at the *p* < 0.05 level.

**Table 2:** Comparison of dietary intake and the Dietary Inflammatory Index between participants who contracted and those who did not contract COVID-19.

Variable	Total *N* = 95	Have Not Contracted COVID-19 *N* = 71	Have Contracted COVID-19 *N* = 24	
	Me (Q1–Q3)	Me (Q1–Q3)	Me (Q1–Q3)	U Mann-Whitney Test *p*
Energy (kcal)	2209 (1897–2478)	2274 (2001–2549)	2120 (1731–2246)	**0.0173**
Water (mL)	2500 (2064–3132)	2680 (2232–3203)	2118 (1671–2681)	**0.0015**
Total protein (g)	82.1 (69.1–103.5)	89 (70.9–109)	77.3 (67.2–88.6)	0.1454
Animal protein (g)	44.7 (24.7–61.8)	44.7 (16.7–64.9)	44.4 (32.6–56.5)	0.6194
Plant protein (g)	37.9 (29.6–47.2)	39.3 (33.3–54.5)	30.3 (25–38.8)	**0.0015**
Arginine (mg)	4621 (3342–5650)	4850 (3650–5716)	3699 (3147–4907)	0.0540
Fat (g)	69.7 (56–85.7)	72.1 (56.4–88.8)	66 (52.8–78.4)	0.2843
Linoleic acid LA (C18:2)	9.8 (7.3–13.7)	10.3 (7.5–14)	8.4 (6.9–12)	0.1576
α-Linolenic acid ALA (C18:3)	1.6 (1.2–2.4)	1.6 (1.3–2.5)	1.5 (1.1–2.4)	0.5041
Omega−3 fatty acids	1.7 (1.1–2.5)	1.7 (1.2–2.3)	1.6 (1–2.6)	0.6561
Omega−6 fatty acids	8 (6.4–11.5)	8.3 (6.5–11.6)	7.6 (5.4–10.2)	0.3639
Total carbohydrates (g)	296.3 (246.1–335.7)	307.9 (265.4–344.7)	272 (220.2–301.3)	**0.0177**
Saccharose (g)	40.1 (30–55.9)	42 (30–55.9)	36 (29–56)	0.7578
Dietary fiber (g)	24.6 (19.1–34.5)	26.8 (22.2–38.1)	18.8 (17–27)	**0.0004**
Alcohol (g)	6 (0–13.2)	6.2 (0–16.5)	5 (0–11.3)	0.6358
Potassium (mg)	3646 (3008–4409)	3758 (3166–4572)	3131 (2509–3777)	**0.0015**
Calcium (mg)	843 (686–1007)	888 (686–1073)	758 (687–884)	0.1252
Magnesium (mg)	410.3 (338.9–548.1)	439.4 (352.8–581.2)	346.9 (285.1–429.7)	**0.0003**
Iron (mg)	15.7 (12.4–19.6)	17 (12.8–20.8)	12.7 (11.1–16.4)	**0.0010**
Zinc (mg)	12.2 (9.5–14.5)	12.6 (9.9–15.2)	10.1 (8.8–12.9)	**0.0248**
Copper (mg)	1.7 (1.3–2.3)	1.8 (1.4–2.4)	1.4 (1.1–1.7)	**0.0004**
Manganese (mg)	6.4 (4.5–8.9)	6.8 (4.8–9.3)	4.8 (3.9–6.3)	**0.0072**
Vitamin A (µg)	1190 (804.3–1532.1)	1209 (863.1–1593.4)	938 (705.3–1496.2)	0.1055
Beta-carotene (µg)	4170 (2889–6481)	4375 (3186–6802)	2924 (2017–5976)	0.0607
Vitamin E (alpha-tocopherol equivalent) (mg)	12.5 (9–16.2)	13.4 (10.3–16.3)	9.1 (7.8–11.9)	**0.0034**
Thiamin (mg)	1.4 (1–1.7)	1.4 (1.2–1.8)	1.2 (0.9–1.4)	**0.0099**
Riboflavin (mg)	1.8 (1.4–2.1)	1.9 (1.5–2.2)	1.6 (1.4–1.9)	0.0821
Niacin (mg)	18.7 (14.9–24.7)	19.2 (15.4–26.6)	16.8 (14.2–20.1)	0.1074
Vitamin B6 (mg)	2 (1.5–2.6)	2.1 (1.6–2.8)	1.8 (1.4–2.2)	**0.0112**
Vitamin B12 (µg)	3.5 (2.6–5.8)	3.6 (2.5–6.1)	3.1 (2.7–4.7)	0.5096
Vitamin C (mg)	109.8 (71.1–162.5)	120 (79.3–171)	107 (47.8–141.7)	0.1111
Folates (µg)	370.1 (274.9–477.8)	392 (297.8–494.1)	279.9 (221.7–378.5)	**0.0024**
Vitamin D (µg) Dietary Inflammatory Index	3.6 (2–8) −0.82 (−2.1–0.93)	3.5 (2–7.8) −1.08 (−2.28–0.45)	4.2 (1.8–11.2) 0.98 (−1.78–1.83)	0.7063 **0.0042**

*N*—number of participants; Me—median; Q1 and Q3—lower and upper quartile. Bold values denote statistical significance at the *p* < 0.05 level.

**Table 3:** Percentage of reference intake for individual macronutrients, vitamins, and minerals in the whole study group and according to the history of COVID-19.

Variable	Total *N* = 95	Have Not Contracted COVID-19 *N* = 71	Have Contracted COVID-19 *N* = 24	
	Me (Q1–Q3) [%]	Me (Q1–Q3) [%]	Me (Q1–Q3) [%]	U Mann-Whitney Test *p*
Water	100.4 (83.2–131.7)	110.9 (89.7–143.9)	85.8 (73.2–107.2)	**0.0011**
Total protein	120.2 (102.6–147.4)	124.9 (105.1–154.3)	112.5 (102.6–129.8)	0.2174
Digestible carbohydrates	206.2 (174.7–231.2)	212.5 (187.8–239.3)	187.5 (153.1–214.1)	**0.0323**
Potassium	104.2 (86–126)	107.4 (90.5–130.6)	89.5 (71.7–107.9)	**0.0015**
Calcium	84.3 (68.7–100.7)	88.8 (68.7–107.3)	75.8 (68.7–88.4)	0.1252
Magnesium	105.9 (87.2–135.7)	110.2 (93.4–147)	86.2 (74.8–105.1)	**0.0001**
Iron	151.7 (108.4–183.2)	156.9 (113.8–202.4)	122.3 (101.3–135.9)	**0.0052**
Zinc	115.2 (93.3–137.6)	123 (97.8–143.9)	94.4 (89.4–118.5)	**0.0088**
Copper	187.6 (147.7–250.3)	198.1 (156.4–270.4)	152.9 (124–189.1)	**0.0004**
Manganese	280 (207.1–405.8)	309.9 (218.5–478.5)	215.6 (191.1–275.1)	**0.0065**
Vitamin A	136.8 (97.9–177)	143.9 (106.3–180.7)	104.2 (88.9–169)	0.0883
Vitamin E (alpha-tocopherol equivalent)	126.5 (93.7–169.4)	139.2 (103.2–178.2)	99.8 (86–118.8)	**0.0042**
Thiamin	107.1 (87.6–138)	112.5 (91.1–143.3)	91.2 (73.9–111.2)	**0.0112**
Riboflavin	137.4 (118.8–169.1)	143.3 (119.7–180.8)	130 (108.5–147.6)	0.0734
Niacin	117.4 (97–159.5)	131.3 (101.2–166.3)	108 (90.8–125.7)	0.0966
Vitamin B6	152.8 (118.3–196.8)	163.2 (123.6–213.3)	135.8 (105.4–170.9)	**0.0112**
Vitamin C	132.9 (79–210.3)	138.9 (90.5–215.3)	118.9 (60.9–160.5)	0.1150
Saturated fatty acids	141.5 (98–180.6)	142.1 (97.9–180.6)	139.1 (109.9–181.6)	0.7906
Linoleic acid LA (C18:2)	78.3 (59.3–102.5)	82.9 (61.9–103.9)	68.7 (59.1–95.9)	0.2441
α-Linolenic acid ALA (C18:3)	101.3 (79.7–159)	103.1 (80.1–159.5)	101.2 (74–155.4)	0.7906
Dietary fiber	98.3 (76.2–137.8)	107.3 (88.7–152.4)	75.3 (68–108.1)	**0.0004**
Folates	92.5 (68.7–119.4)	98 (74.4–123.5)	70 (55.4–94.6)	**0.0024**
Vitamin B12	146 (109.4–242.2)	149.7 (106.2–254.9)	128.8 (111–194.1)	0.5096
Vitamin D	23.8 (13.2–53.5)	23.3 (13.2–51.9)	28.1 (12.3–74.7)	0.7063

*N*—number of participants; Me—median; Q1 and Q3—lower and upper quartile. Bold values denote statistical significance at the *p* < 0.05 level.

**Table 4:** Consumption of selected food products by the respondents.

Variable		Total *N* = 95		Have Not Contracted COVID-19 *N* = 71		Have Contracted COVID-19 *N* = 24		
		Me (Q1–Q3)		Me (Q1–Q3)		Me (Q1–Q3)		U Mann-Whitney Test *p*
Groats and rice (g)		14.3 (6.5–29.6)		18 (9.9–33.3)		8.8 (0–22.8)		**0.0129**
Seeds (g)		2 (0–6.7)		2.5 (0–6.9)		1.4 (0–4.3)		0.2686
Nuts (g)		13.7 (2.2–30.8)		16.3 (6.7–35.6)		1.9 (0.5–16.1)		**0.0035**
Fruit (g)		204.6 (111.4–400.4)		231.4 (110.8–419.5)		154 (115.8–282.8)		0.2511
Vegetable (g)		343.3 (236.1–470.9)		361 (262.5–476.5)		240.8 (167.6–414.8)		**0.0136**
Fruit and vegetables (g) Legumes (g)		554.9 (409.9–781.8) 10 (0–50.9)		579,1 (455.3–839.4) 16 (1.5–72.8)		431.8 (262.6–670.3) 2.4 (0–11.4)		**0.0177** **0.0125**
**Variable**	**Category**	*N*	%	*N*	%	*N*	%	**Chi^2^** ***p***
Garlic	<1 g on average daily	78	82.1	55	77.5	23	95.8	**0.0424**
	≥1 g on average daily	17	17.9	16	22.5	1	4.2	
Onion	<10 g on average daily	70	73.7	50	70.4	20	83.3	0.2143
	≥10g on average daily	25	26.3	21	29.6	4	16.7	
Fruit and vegetables	500 g and more per day	59	62.1	48	67.6	11	45.8	0.0573
	Less than 500 g per day	36	37.9	23	32.4	13	54.2	
Nuts	10 g and more per day	56	58.9	48	67.6	8	33.3	**0.0032**
	Up to 10 g per day	39	41.1	23	32.4	16	66.7	

*N*—number of participants; Me—median; Q1 and Q3—lower and upper quartile. Bold values denote statistical significance at the *p* < 0.05 level.

**Table 5:** The odds ratio for the relationship between the consumption of vegetables, fruits, and nuts and developing COVID-19.

Model	FV ≥ 500 g and nuts ≥ 10 g	Failure to Meet One of the Conditions	FV< 500 g and Nuts < 10 g	*p*_trend_
**Model 1 ^a^**	1 (ref.)	4.13 (1.25–16.30)	7.36 (2.00–31.81)	0.003
**Model 2 ^b^**	1 (ref.)	5.03 (1.39–21.97)	9.01 (1.99–49.31)	0.005
**Model 3 ^c^**	1 (ref.)	5.73 (1.49–27.82)	12.22 (2.47–78.12)	0.003

^a^—crude model; ^b^—adjusted to type of diet, sex, marital status, age, BF%, and smoking; ^c^ adjusted to covariates included in b and additionally to bacteria *Klebsiella* spp.; FV—fruit and vegetables.

**Table 6:** Dietary Inflammatory Index and consumption of selected food products by respondents divided according to compliance with the recommended consumption of vegetables, fruit, and nuts.

Variable		Average Daily Consumption of >500 g of Fruit and Vegetables and >10 g of Nuts *N* = 40		Failure to Meet One of the Conditions (Daily Consumption of <500 g of Fruit and Vegetables or <10 g of Nuts) *N* = 35		Failure to Meet any of the Conditions (Daily Consumption of <500 g of Fruit and Vegetables and <10 g of Nuts) *N* = 20		
		Me (Q1–Q3)		Me (Q1–Q3)		Me (Q1–Q3)		Kruskal–Wallis Test *p*
Groats and rice (g)		21.6 (8.6–42.5)		13 (1–28)		12.2 (7.1–22.4)		0.1655
Seeds (g)		4.1 (1.5–9.6)		1.7 (0–8.7)		0 (0.0–1.3)		**0.0001**
Nuts (g)		28.7 (17.8–46.9)		9.2 (2.2–17.3)		0.9 (0.0–2.1)		**<0.0001**
Fruit (g)		332.4 (237.8–442.4)		167.8 (110.8–347.9)		105.3 (39.8–124)		**<0.0001**
Vegetable (g)		427 (354.3–509.7)		305.2 (212.2–454.5)		233.9 (130.3–297.5)		**<0.0001**
Fruit and vegetables (g) Legumes (g) Dietary Inflammatory Index		722.6 (587.1–971.6) 25.8 (6.2–78.9) −1.77 (−2.78–0.98)		521.2 (420.5–698.5) 6.3 (0.0–30.7) 0.25 (−2.08–0.93)		354.9 (231.2–386.5) 2.1 (0.0–14.9) 1.46 (0.23–2.93)		**<0.0001** **0.0137** **<0.0001**
**Variable**	**Category**	***N***	**%**	***N***	**%**	***N***	**%**	**Chi^2^** ***p***
Garlic	<1 g on average daily	29	72.5	30	85.7	19	95.0	0.0787
	≥1 g on average daily	11	27.5	5	14.3	1	5.0	
Onion	<10 g on average daily	26	65.0	26	74.3	18	90.0	0.1160
	≥10 g on average daily	14	35.0	9	25.7	2	10.0	
Fruit and vegetables	500 and more g per day	40	100.0	19	54.3	0	0.0	**<0.0001**
	<500 g per day	0	0.0	16	45.7	20	100.0	
Nuts	10 and more g per day	40	100.0	16	45.7	0	0.0	**<0.0001**
	Up to 10 g per day	0	0.0	19	54.3	20	100.0	
Diet	Traditional	18	45.0	20	57.1	16	80.0	**0.0358**
	Vegetarian	22	55.0	15	42.9	4	20.0	

*N*—number of participants; Me—median; Q1 and Q3—lower and upper quartile. Bold values denote statistical significance at the *p* < 0.05 level.

## Data Availability

The data presented in this study are not publicly available due to confidentiality reasons. These data are available on request from the corresponding author.

## Supplementary Materials

The following are available online at https://www.mdpi.com/article/10.3390/nu14020350/s1,: Table S1: Example of traditional menus., Table S2: Examples of vegetarian menus.
