# Supplementary material for: Associations of Nutritional Behavior and Gut Microbiota with the Risk of COVID-19 in Healthy Young Adults in Poland
Source: Nutrients. 2022 Jan 14;14(2):350. doi: 10.3390/nu14020350 (PMC8779092; doi:10.3390/nu14020350)
Supplement: Supplementary file 1 [file nutrients-14-00350-s001.zip › nutrients-1545164-supplementary.pdf]

nutrients MDPI

Table S1. Example of traditional menus.

Average daily consumption of >500 g of fruit and vegetables and >10 g of nuts Have not contracted COVID-19			Failure to meet any of the conditions Have contracted COVID-19		
**Meal**	**Product / dish**	**Amount [g/ml]**	**Meal**	**Product / dish**	**Amount [g/ml]**
I breakfast	Oat flakes	46	I breakfast	Plain bread	100
	Skimmed milk powder	7		Tomato	100
	White chocolate	5		Smoked mackerel	50
	Strawberry yogurt 1.5% fat	350		Sandwich ham	50
	Tea, sugar-free	200		Tea, sugar-free	250
II breakfast	Graham rolls	50		Yeast cakes with strawberries	150
	Tuna in water	30		Coffee, sugar-free	500
	Canned corn	30		Food milk, 2% fat	100
	Carrot	30	II breakfast	Tea, sugar-free	1200
	Green peas, cooked	30			
	Pickled green olives	30	Lunch	Broth	200
	Orange juice	500		Cooked pasta	100
Lunch	Pork goulash	150		Breaded chicken breast cutlet	100
	Buckwheat groats	60		Potatoes	70
	Tomato soup made from fresh tomatoes	250		Lettuce	20
				Cream 12% fat	10
	Carbonated sugar sweetened drinks	250		Banana	20
Dinner	Vegetables for the pan (broccoli 120 g, corn 120g, Carrot 120 g, sugar peas 90 g)	450	Dinner	Plain bread	150
	Tran Moller's Lemon Flavored - (Peter Moller)	5		Sandwich ham	50
				Beer full	500
	Tea, sugar-free	250			
	Orange juice	250			

Table S2. Examples of vegetarian menus.

Average daily consumption of >500 g of fruit and vegetables and >10 g of nuts<br>Have not contracted COVID-19			Failure to meet any of the conditions<br>Have contracted COVID-19		
**Meal**	**Product / dish**	**Amount [g/ml]**	**Meal**	**Product / dish**	**Amount [g/ml]**
I breakfast	Water	500	I breakfast	Graham bread	70
	Oat flakes	50		Cheese, full fat emmental	30
	Soy milk	250		Tomato	30
	Peanuts	30		Tea, sugar-free	200
	Linseed	10			
	Honey	10	II breakfast	Mineral water „Muszyna"	1000
	Water	200		Cheesecake with raisins	150
	Coffee, sugar-free	200		Yeast buns with poppy seed filling	100
	Soy milk	50	Lunch	Coffee, sugar-free	100
II breakfast	Apple	150		Bean cutlet (no eggs added)	50
	Plums	100		Village bread	30
	Apple juice	200		Beets	30
Lunch	Cauliflower	100		Cheese, full fat emmentaler	30
	Boiled beans	150	Dinner	Tea, sugar-free	200
	Tomato sauce	100		Plain wheat rolls	60
	Boiled chickpeas	100		Butter	5
	Graham bread	120		Beets	30
Snack	Water	200		Water	200
	Blueberry yogurt 1.5% fat	140			
Dinner	Graham bread	80			
	"Feta" type cheese	20			
	Fried eggs	120			
	Butter lettuce	20			
	Olive oil	10			
	Cucumber	100			
